# Combining immunosuppressive therapy with low dosage eltrombopag in Chinese patients with severe aplastic anemia: mild aggravation of hepatic injury

**DOI:** 10.1007/s00277-025-06210-7

**Published:** 2025-02-05

**Authors:** Xiaoyu Chen, Qingling Yu, ChengTao Qin, Yawen Zhang, Jingnan Sun, Jinsong Jia, Baodong Ye, Yuemin Gong, Guangsheng He, Lei Fan

**Affiliations:** 1 Department of Hematology, Collaborative Innovation Center for Cancer Personalized Medicine, The First Affiliated Hospital of Nanjing Medical University, Jiangsu Province Hospital, Key Laboratory of Hematology of Nanjing Medical University, Nanjing, China; 2 Department of Hematology, Affiliated Jianhu Hospital of Nantong University Xinglin College, Yancheng, China; 3 Department of Hematology, The First Hospital of Jilin University, Changchun, China; 4 Department of Hematology, Peking University People’s Hospital, Peking University Institute of Hematology, Beijing, China; 5 Department of Hematology, Zhejiang Province Hospital of Traditional Chinese Medicine, The First Affiliated Hospital of Zhejiang Chinese Medical University, Hangzhou, China

**Keywords:** Hepatic injury, Eltrombopag, Immunosuppressive therapy, Severe aplastic anemia

## Abstract

Eltrombopag (EPAG) is an oral thrombopoietin receptor agonist analog with the potential risk to induce liver injury. This prospective registry study evaluated the prevalence and severity of hepatic injury in Chinese patients with severe aplastic anemia undergoing low-dose EPAG treatment (75 mg/day) in the context of standard immunosuppressive therapy (IST). The incidence of acute drug-induced liver injury was slightly higher in the IST + EPAG group than in the IST group at the 1st and 2nd month, but no statistically significant difference was observed: 10% vs 5% (*p* = 0.400), 9% vs 8% (*p* = 1.000). At the 1st month, the incidences of alanine aminotransferase, aspartate aminotransferase, and total bilirubin increased of grade 3 or higher in the IST + EPAG and the IST groups, were 5% vs 3% (*p* = 0.228), 2% vs 1% (*p* = 1.000), 2% vs 1% (*p* = 1.000), respectively. The logistic analysis indicated that serum ferritin level was associated with severe liver injury events. There was a slight increase in the incidence of severe hepatic injury events in the patients with SAA treated by EPAG, but it was insignificant.

## Introduction

Aplastic anemia is a bone marrow failure disorder caused mainly by abnormal autoimmunity. It is classified as either severe aplastic anemia (SAA) or non-severe aplastic anemia depending on the degree of cytopenia [1]. Immunosuppressive treatment (IST) with eltrombopag (EPAG) is recommended for patients with SAA who are not eligible for allogeneic hemopoietic stem cell transplant from a matched sibling donor [2].

EPAG is a small-molecule oral thrombopoietin receptor (TPO-R) agonist analog that binds to the transmembrane region of TPO-R and promotes the expansion of hematopoietic stem and progenitor cells [3, 4]. It is primarily metabolized by hepatic cytochrome P450 (CYP) 1A2, CYP2C9 isoenzymes, uridine diphosphate-glucuronosyltransferase (UGT) 1A1 and UGT1A3 in vivo, which poses a risk of hepatic injury [5]. Therefore, the United States Food and Drug Administration added a black box warning for EPAG that it may increase the risk of serious or potentially life-threatening hepatotoxicity. In chronic immune thrombocytopenia (ITP) patients, hepatic injury of higher incidence and severity was observed in the EPAG group compared to the placebo group [6, 7]. Hepatic adverse events have also been reported in single-arm clinical trials of EPAG monotherapy in refractory SAA [8, 9]. Owing to the lower EPAG clearance in Asian people, the dose of EPAG for Asian patients should be approximately half that recommended for other populations [10]. Hepatobiliary laboratory abnormalities were reported in about 10% of Chinese ITP patients in a randomized clinical trial [11]. The incidence was even higher (22.4%) in a real-world retrospective study [12]. SAA patients receiving IST + EPAG treatment need to take EPAG at a higher dosage and for a longer duration than ITP patients, and usually in combination with other drugs having potential hepatotoxicity, e.g. cyclosporine. However, the profile of EPAG-induced liver injury in Chinese SAA patients receiving combined treatment of IST and EPAG is yet unclear.

This study aims to investigate the incidence and severity of liver injury in Chinese patients with SAA treated with EPAG in the context of standard IST in the real world.

## Methods

### Patients

We recruited 145 adult SAA patients from October 2014 to September 2023 in a prospective registry study (ChiCTR2100045895) in the Chinese Eastern Collaboration Group of Anemia (CECGA), which includes the First Affiliated Hospital of Nanjing Medical University, the First Hospital of Jilin University, Peking University People’s Hospital, Zhejiang Provincial Hospital of Chinese Medicine and Tongji Hospital of Tongji Medical College of Huazhong University of Science and Technology. Informed consent was obtained in accordance with the principles outlined in the Declaration of Helsinki. This study received approval from the ethics committees of the participating hospitals. Inclusion criteria included (1) diagnosed with SAA per Camitta criteria[2], (2) receiving rabbit anti-thymocyte globulin (r-ATG) plus cyclosporine-based IST as first-line therapy, (3) age ≥ 18 years old, (4) complete liver function monitoring data before and after IST treatment, and (5) oral administration of EPAG for at least 6 months in patients treated with EAPG. Individuals with chronic hepatitis B virus (HBV) infection [defined as hepatitis B virus surface antigen (HBsAg) seropositive status beyond 6 months[13] or other chronic liver diseases were excluded.

Based on the treatment regimens, they were categorized into the IST + EPAG group (63 patients) and the IST alone group (82 patients). Liver function indices such as alanine aminotransferase (ALT), aspartate aminotransferase (AST), alkaline phosphatase (ALP), gamma-glutamyl transpeptidase (GGT), and total bilirubin (TBIL) were dynamically monitored for 6 months following r-ATG administration. The incidence of elevated liver function indices in both groups was calculated on a monthly basis. The severity was graded according to the drug-induced liver injury (DILI) [14] and the 2017 National Cancer Institute Common Terminology Criteria for Adverse Events (CTCAE v5.0) [15]. DILI is classified according to the pattern of liver tests observed – hepatocellular, cholestatic, or mixed. Hepatocellular DILI is characterized as ALT ≥ 3 times the upper limit of normal (ULN) and ALT/ ALP ratio ≥ 5 times ULN; cholestatic DILI by an ALP ≥ 2 times ULN and ALT/ALP ratio of ≤ 2 times ULN; and mixed DILI with ALT ≥ 3 times ULN, ALP ≥ 2 times ULN and ALT/ALP ratio < 5 but > 2 times ULN [14].

### IST regimen

R-ATG was intravenously infused at 2.5 ~ 3.5 mg/kg/day for 5 days. Prednisone acetate at a dose of 1 mg/kg/day was given for 2 weeks to prevent allergic reaction and serum sickness, followed by rapid tapering over the next 2 weeks.

Cyclosporine, initially at a dose of 3 mg/kg/day, was administered twice per day orally. After one week, the blood concentration of cyclosporine was measured, and the trough blood concentration was maintained at 150 ~ 250 ng/mL for at least 12 months. The dose was adjusted according to the blood concentration and the occurrence of adverse reactions, and then gradually decreased and plateaued within 6 ~ 12 months according to the hematological indices.

### EPAG

EPAG was initially administered at a dosage of 25 mg daily, which increased by 25 mg every 3 days to 75 mg daily within one week. The final dosage was maintained for at least 6 months. Table 1 shows the dosage adjustment scheme of EPAG according to hematological indices [5].

**Table 1:** Dose adjustment scheme of EPAG

Platelet count > 400 × 10^9^/L	Discontinue EPAG for 1 week. Restart at dosage decreased by 25 mg/day if platelets drop to < 100 × 10^9^/L
Platelet count ≥ 100 × 10^9^/L but ≤ 400 × 10^9^/L	Reduce dosage by 25 mg every 2 weeks to the lowest dosage that maintains platelet count ≥ 50 × 10^9^/L
Platelet count ≥ 50 × 10^9^/L and HB* ≥ 100 g/L without transfusion and ANC* ≥ 1 × 10^9^/L without G-CSF* for 8 weeks	Reduce dosage by 50%
After decreased dosage, platelet count ≥ 50 × 10^9^/L and HB ≥ 100 g/L without transfusion and ANC ≥ 1 × 10^9^/L without G-CSF for another 8 weeks	Discontinue EPAG
After decreased dosage, platelet count < 50 × 10^9^/L or HB < 90 g/L or ANC < 0.5 × 10^9^/L	Restart EPAG or the previous effective dosage

* *EPAG* Eltrombopag,; *HB* Hemoglobin; *ANC* Absolute neutrophil count, *G-CSF* Granulocyte colony-stimulating factor

### Management of elevated hepatic indices

In cases where patients exhibited abnormal levels of ALT and AST during treatment, silymarin, magnesium isoglycyrrhizinate, bicyclol, or polyene phosphatidylcholine were administered. On the other hand, if ALP, GGT, or TBIL levels were abnormal, adenosylmethionine butane-disulfonate and ursodeoxycholic acid were prescribed. For grade 3 or above liver injury events, cyclosporine and EPAG were tapered and, if necessary, suspended.

### Statistical analysis

The differences in quantitative data were analyzed using the Mann–Whitney U test and qualitative data were assessed using the Chi-square test and Fisher exact probability method with SPSS 26.0 statistical software. The binary logistic regression model was employed to analyze related risk factors for DILI at the 1st month, while the receiver operating characteristic (ROC) curve was used to evaluate the predictors. GraphPad Prism 9 software was utilized to create line and bar graphs and a significance level of *P* < 0.05 was used.

## Results

### Baseline features

The clinical characteristics of 145 adult SAA patients are shown in Table 2. There were no significant differences between IST + EPAG and IST group in age range, gender distribution, body mass index (BMI), serum ferritin level, peripheral blood cell counts and resolved hepatitis B virus (HBV) infection before treatment.

**Table 2:** Comparison of the clinical characteristics of SAA patients in IST + EPAG and IST groups

Variables	IST + EPAG (*n* = 63)	IST (*n* = 82)	*P*
Age/year, Median (Q₁, Q₃)	38.67 (27.04, 56.12)	39.58 (26.96, 55.25)	*0.585*
Gender, *n* (%)			0.700
Male	31 (49.21)	43 (52.44)	
Female	32 (50.79)	39 (47.56)	
BMI, Median (Q₁, Q₃)	23.51 (21.40, 25.51)	23.38 (20.62, 25.45)	0.700
Serum ferritin level ng/ml, Median (Q₁, Q₃)	581.40 (331.40, 1014.03)	755.30 (460.45, 1312.20)	0.120
Cyclosporin serum trough concentration ng/ml, Median (Q₁, Q₃)	192.00 (187.53, 192.00)	192.00 (173.31, 216.44)	0.202
ANC* (× 10^9^/L), Median (Q₁, Q₃)	0.40 (0.20, 0.60)	0.41 (0.21, 0.69)	0.632
ALC* (× 10^9^/L), Median (Q₁, Q₃)	0.96 (0.65, 1.38)	1.04 (0.57, 1.46)	0.949
LY%*, Median (Q₁, Q₃)	61.30 (47.00, 71.90)	62.10 (35.45, 74.70)	0.992
RBC* (× 10^12^/L), Median (Q₁, Q₃)	1.88 (1.65, 2.24)	1.96 (1.71, 2.22)	0.551
HB* (g/L), Median (Q₁, Q₃)	60.00 (55.00, 70.00)	61.50 (56.00, 69.75)	0.641
APC* (× 10^9^/L), Median (Q₁, Q₃)	9.00 (5.00, 13.00)	11.50 (7.00, 17.00)	0.059
ARC* (× 10^9^/L), Median (Q₁, Q₃)	17.33 (9.79, 35.35)	13.72 (6.47, 24.03)	0.060
^Resolved HBV infection, n (%)	2 (3.33)	1 (1.25)	0.800

*IST Immunosuppressive therapy; *EPAG* Eltrombopag; *BMI* Body mass index; *ANC* Absolute neutrophil count; *ALC* Absolute lymphocyte count, *LY%* Percentage of lymphocytes; *RBC* Red blood cell count; *HB* Hemoglobin; *APC* Absolute platelet count; *ARC* Absolute reticulocyte count; *HBV* Hepatitis B virus; *Q* Quartile. ^ HBsAg(-) and anti-HBs(+) [13]

### Comorbidities

In the IST + EPAG group, there were six individuals diagnosed with hypertension, three diagnosed with diabetes, one with rheumatic disease, one with hypothyroidism, and one with chronic nephritis, vitiligo, and syphilis. In the IST group, there were five cases of hypertension, four cases of cardiac system disease, 3 cases of diabetes, one case of rheumatic disease, one case of asthma, and one case of pityriasis rosea.

### Elevated hepatic indices of any grade

**Fig. 1:**
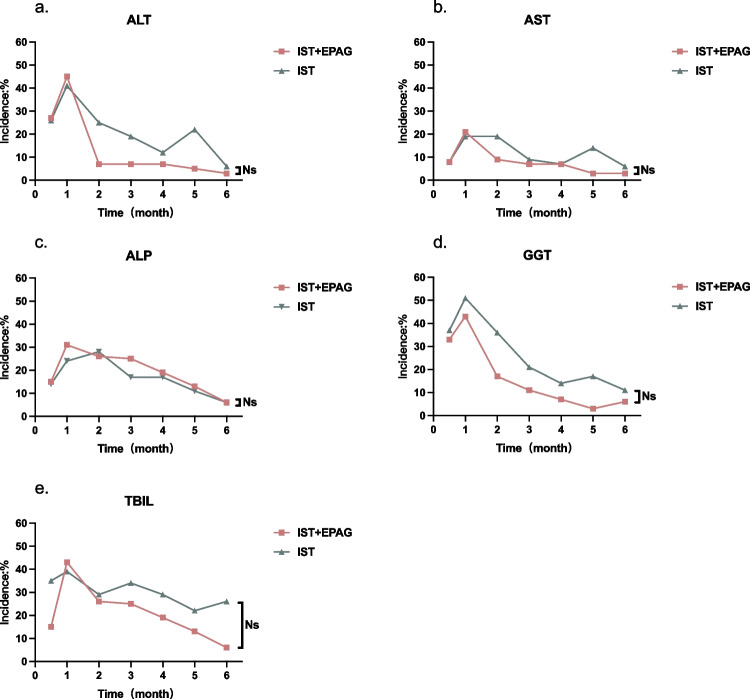
The abnormalities of **ALT (1-a)**, **AST (1-b)**, **ALP (1-c)**, **GGT (1-d)**, and **TBIL (1-e)** were compared monthly after using r-ATG in the IST + EPAG and IST alone groups. The difference between two groups at each time point is of no statistical significance (ns) (*p* > 0.05). *ALT alanine aminotransferase, AST aspartate aminotransferase, ALP alkaline phosphatase, GGT gamma-glutamyl transpeptidase, TBIL total bilirubin, IST immunosuppressive therapy, EPAG eltrombopag

The elevation of ALT, AST, ALP, GGT, and TBIL mostly occurred within the 1st month after r-ATG treatment (Fig. 1, and Table 3). After treatment, liver injury gradually improved. The median duration for the elevation of ALT, AST, ALP, GGT, and TBIL levels in the two groups was: 1.5 (1–3) vs 2.5 (1–5) weeks, 2 (1–4) vs 2.5 (1–5) weeks, 3 (2–8) vs 4.5 (1–8) weeks, 5 (1–8) vs 4 (1–12) weeks and 4 (1–9) vs 4.5 (1–7) weeks, respectively. Within 6 months after receiving r-ATG, no significant difference was observed between the two groups in terms of the incidence or the duration of liver dysfunction.

**Table 3:** The incidence and duration of abnormal liver function indices in the IST + EPAG and IST alone groups

		1st m	2nd m	3rd m	4th m	5th m	6th m	median duration (weeks)
ALT *n* (%)	IST + EPAG	26 (45)	3 (7)	3 (7)	3 (7)	2 (5)	1 (3)	1.5 (1–3)
	IST	32 (41)	17 (25)	10 (19)	5 (12)	8 (22)	2 (6)	2.5 (1–5)
AST *n* (%)	IST + EPAG	12 (21)	4 (9)	3 (7)	3 (7)	1 (3)	1 (3)	2 (1–4)
	IST	15 (19)	13 (19)	5(9)	3 (7)	5 (14)	2 (6)	2.5 (1–5)
ALP *n* (%)	IST + EPAG	18 (31)	12 (26)	11 (25)	8 (19)	5 (13)	2 (6)	3 (2–8)
	IST	19 (24)	19 (28)	9 (17)	7 (17)	4 (11)	2 (6)	4.5 (1–8)
GGT *n* (%)	IST + EPAG	25 (43)	8 (17)	5 (11)	3 (7)	1 (3)	2 (6)	5 (1–8)
	IST	40 (51)	25 (36)	11 (21)	6 (14)	6 (17)	4 (11)	4 (1–12)
TBIL *n* (%)	IST + EPAG	25 (43)	12 (26)	11 (25)	8 (19)	5 (13)	2 (6)	4 (1–9)
	IST	31 (39)	20 (29)	18 (34)	12 (29)	8 (22)	9 (26)	4.5 (1–7)

*ALT Alanine aminotransferase; *AST* Aspartate aminotransferase; *ALP* Alkaline phosphatase; *GGT* Gamma-glutamyl transpeptidase; *TBIL* Total bilirubin; *IST* Immunosuppressive therapy; *EPAG* Eltrombopag

### DILI

**Fig. 2:**
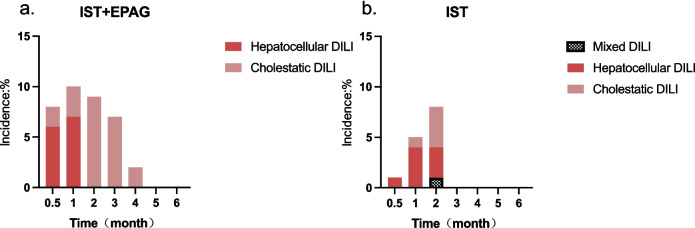
A comparative analysis of the incidence of DILI over time in the **IST + EPAG (2-a)** and **IST alone (2-b)** groups showed no significant differences. *DILI drug-induced liver injury, IST immunosuppressive therapy, EPAG eltrombopag

In the IST + EPAG group, all hepatocellular and cholestatic DILI events occurred within 4 months after r-ATG. DILI events were observed within 2 months after r-ATG in the IST group, including hepatocellular, cholestatic, and mixed types. Hepatocellular DILI was the predominant type in the 1st month. The incidence of DILI was slightly higher in the IST + EPAG group than in the IST group in the 1st and 2nd month, but there was no statistically significant difference: 6 (10%) vs 4 (5%) (*p* = 0.400), 4 (9%) vs 6 (8%) (*p* = 1.000). DILI still occurred in the IST + EPAG group in the 3rd (3 patients) (7% vs 0%, *p* = 0.088) and 4th (1 patient) months (2% vs 0%, *p* = 1.000), all of which being cholestatic (Fig. 2).

**Fig. 3:**
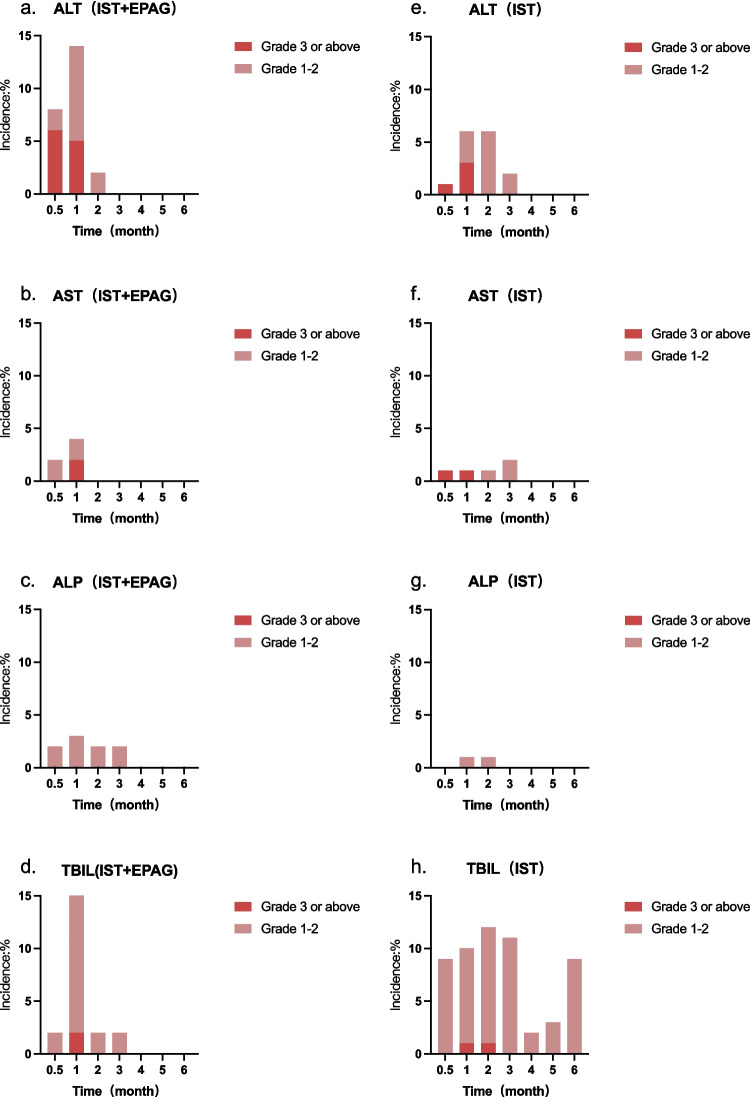
There was no significant difference in the changing trend of ALT, AST, ALP, and TBIL between the IST + EPAG **(3-a, 3-b, 3-c, 3-d)** and IST alone **(3-e, 3-f, 3-g, 3-h)** groups under CTCEA classification. *ALT alanine aminotransferase, AST aspartate aminotransferase, ALP alkaline phosphatase, TBIL total bilirubin, IST immunosuppressive therapy, EPAG eltrombopag

The severity of the elevation in ALT, AST, ALP, and TBIL in the two groups was evaluated (Fig. 3) by CTCAE v5.0. Grade 3 or above adverse events of ALT, AST, and TBIL in the IST + EPAG group and the IST group, were 3 (5%) vs 2 (3%) (*p* = 0.228), 1 (2%) vs 1 (1%) (*p* = 1.000), 1 (2%) vs 1 (1%) (*p* = 1.000), at the 1st month, respectively. One patient in the IST group still had a grade 3 adverse event of TBIL (0% vs 1%, *p* = 1.000) in the 2nd month. Four patients in the IST + EPAG group had EPAG treatment halted, and cyclosporine dose reduced due to a grade 3 hepatic impairment event. One patient with a grade 3 ALT abnormality event in the IST arm had the cyclosporine dosage reduced, and one had the cyclosporine administration discontinued because of a grade 4 ALT abnormality event. The original regimen was resumed after the recovery of the liver function indices.

### Risk factors of DILI

**Fig. 4:**
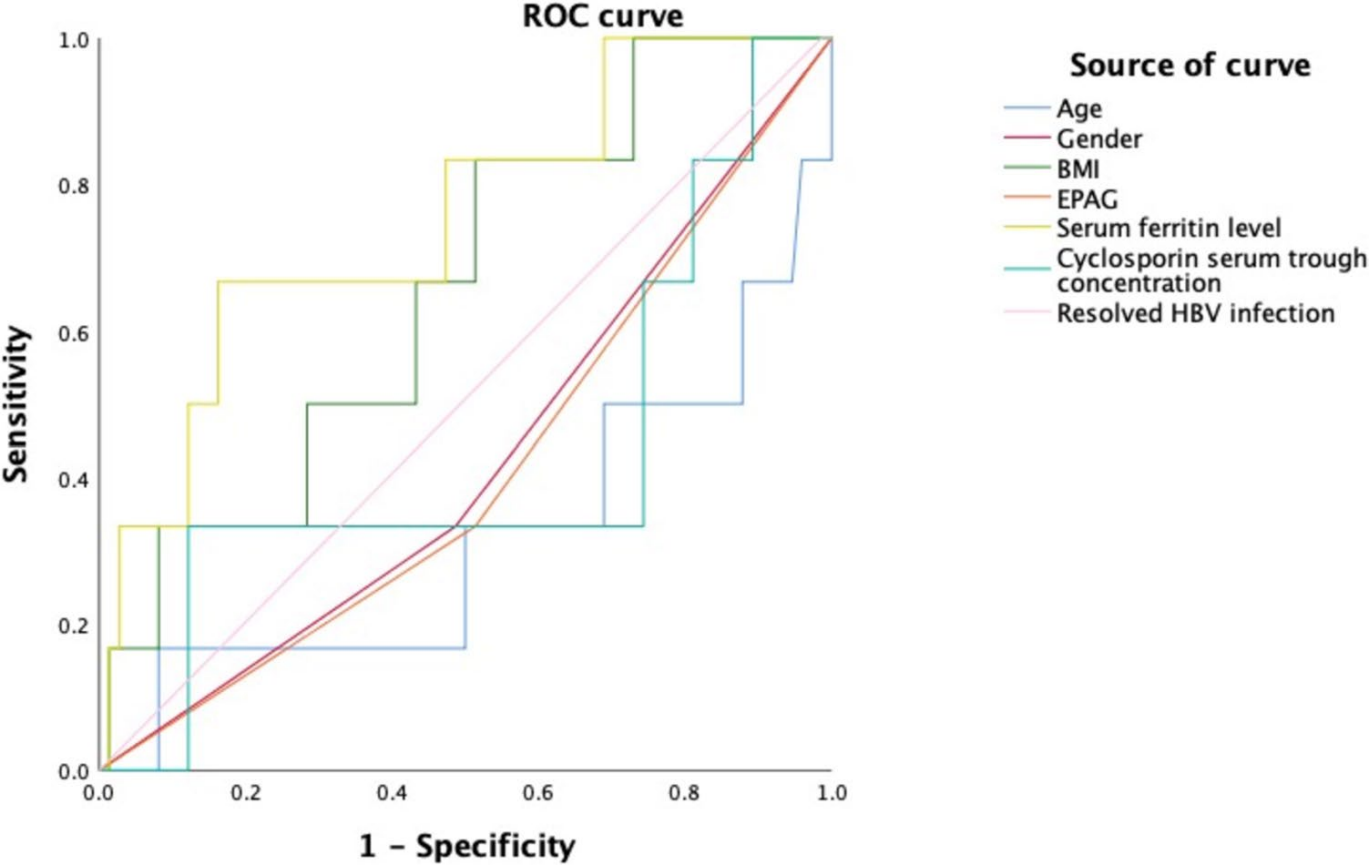
The receiver operating characteristic (ROC) curve was employed to identify factors predicting DILI at the 1st month, with an Area Under the Curve (AUC) for serum ferritin level exceeding 0.7. * EPAG eltrombopag, BMI body mass index, HBV hepatitis B virus

The risk factors of DILI of 145 SAA patients at the 1st month were analyzed. Univariate and multivariate logistic analysis showed that serum ferritin level was a contributing factor for DILI (Table 4). Patients with DILI had a higher serum ferritin level (median: 1329.30 ng/ml vs 678.75 ng/ml). The ROC curve was used to evaluate the predictors for DILI at the 1st month. Area Under the Curve (AUC) of age, gender, BMI, EPAG, resolved hepatitis B infection, and serum ferritin level was 0.316, 0.423, 0.658, 0.410, 0.529, and 0.752, respectively. The optimal threshold of serum ferritin level was 1204.45 ng/ml at the maximum of the Youden index. The sensitivity and specificity of serum ferritin level were 66.7% and 83.8%. The pretreatment factors including age, gender, BMI, EPAG, cyclosporin serum trough concentration and resolved HBV infection were not found to be predictive of DILI at the 1st month (Fig. 4).

**Table 4:** Univariate and multivariate analysis of the occurrence of DILI in the first month

Category and variable	*P* value	
	Univariate	Multivariate
Age	0.555	0.471
Gender	0.559	0.561
BMI	0.128	0.169
EPAG	0.282	0.341
Serum ferritin level	**0.006**	**0.046**
Cyclosporin serum trough concentration	0.103	0.740
Resolved HBV infection	0.992	0.995

## Discussion

EPAG is an orally available TPO-R non-peptide agonist with a carboxylic acid (COOH) group on one end, a lipophilic (CH_3_) groups on the other, and a metal chelate group in the middle. EPAG treatment is considered a risk factor for liver damage in patients with ITP [12]. Liver damage also occurred in some patients with refractory aplastic anemia during EPAG monotherapy [9]. The EPAG clearance is 33–52% lower in Asian patients than in other populations. Therefore, the starting dose of EPAG for Asian patients should be recommended to about 50% of that of other populations [10]. Therefore, we investigated the specific situation of liver injury in Chinese SAA patients receiving low-dose EPAG treatment in the real world.

Compared with the data reported in Chinese ITP patients, hepatobiliary laboratory abnormalities of any grade were actually more frequent in our IST + EPAG cohort (45% at peak vs 10% ~ 22%) [11, 12], probably due to higher EPAG dosage and combination medication. The abnormal ALT, AST, ALP, GGT, and TBIL levels in IST + EPAG and IST alone groups peaked within the first month following IST and declined gradually. Liver injury ≥ grade 3 occurred mainly within 2 months after r-ATG treatment. This may be caused by early prophylactic antifungal treatment and the increased iron burden of frequent blood product transfusions [2]. The incidence of severe liver injury in the IST + EPAG group was higher than in the control group. The period of DILI in SAA patients treated with EPAG was longer, and DILI was still present in the third and fourth months, all of which being cholestatic types. It suggested that EPAG may aggravate the existing liver dysfunction and potentially lead to damage of the bile duct system.

We found that a high baseline serum ferritin level was a risk factor for severe liver damage. SAA patients with high RBC transfusion burden frequently exhibit iron overload. They are at high risk of developing liver damage since the liver is one of the primary organs targeted for iron overload injury [16]. However, this retrospective study was limited in exploring a cause-effect link between hyperferritinaemia and high susceptibility to liver injury after IST.

EPAG contains a metal-chelating structure capable of sequestering iron ions in the body and ameliorating iron overload [17]. Additionally, EPAG reduces transfusion burden and mitigates further iron deposition in the liver [18]. It facilitates hematopoietic recovery and enhances the utilization of stored iron and ultimately mitigates liver injury from iron overload. This may explain why EPAG does not significantly exacerbate liver injury in patients with aplastic anemia.

In the IST + EPAG regimen, the recommended duration of EPAG treatment is 6 months. The real-world multi-center study from CECGA shows that extending the duration of EPAG can continue to benefit patients with partial response and no response [19]. Due to ethnic differences in drug metabolism, the recommended dose of EPAG in Asians is 75 mg/d [10]. Further exploration of the maximum tolerated dose and optimal duration of eltrombopag in Chinese SAA patients should place more emphasis on the changing trend of EPAG-related liver damage with time and dose.

## Data Availability

No datasets were generated or analysed during the current study.
